# Risk Factors associated with dysphagia for people with Cervical Spinal Cord Injury (cSCI): A Systematic review and Meta-analysis

**DOI:** 10.1038/s41393-026-01258-2

**Published:** 2026-08-26

**Authors:** Sharon Leigh, Chunhu Shi, Ian Benson, Jackie McRae

**Affiliations:** 1 School of Health Sciences, Faculty of Biology, Medicine and Health, Manchester Academic Health Science Centre, University of Manchester, Jean McFarlane Building, Oxford Rd, Manchester, M13 9PL UK; 2 National Spinal Injuries Centre, Stoke Mandeville Hospital, Mandeville Road, Aylesbury, HP21 8AL UK; 3 NIHR Applied Research Collaboration Greater Manchester (ARC-GM), Manchester, UK; 4 Department of Allied Health, School of Health & Medical Sciences City St George’s, University of London Cranmer Terrace, London, SW17 0RE UK

**Keywords:** Risk factors, Neurophysiology

## Abstract

**Study Design:**

Systematic Review.

**Objectives:**

This systematic review aimed to summarise evidence on the risk factors associated with dysphagia for people with Cervical Spinal Cord Injury (cSCI).

**Setting:**

Studies originate from multiple countries, no single study setting applies.

**Methods:**

We searched Medline and Embase (OVID), CENTRAL, CINAHL (EBSCOhost), Web of Science (Clarivate), ProQuest and Scopus on 16 January 2025 with an update on 27 July 2025 for prospective and retrospective cohort studies, cross-sectional and non-randomised observational longitudinal studies. These studies included patients aged ≥ 16 years with cSCI and no pre-existing oropharyngeal dysphagia or oesophageal dysphagia. Following standard Cochrane review methodology, two reviewers screened the search results and the full text papers of potentially relevant studies independently and then assessed methodological quality using Newcastle Ottawa Scale (NOS). The certainty of evidence was appraised using Grading of Recommendations, Assessments, Development and Evaluations (GRADE). Data were extracted using a piloted tool. Meta-analysis for each risk factor was conducted using RevMan, pooling Odds Ratios (ORs) from multivariate and univariate models.

**Results:**

Eighteen studies (2, 258 participants) were included. Our meta-analyses suggested that three risk factors had significant associations with dysphagia in cSCI: tracheostomy (OR = 4.91, 95% CI: 1.74 to 13.86, *p* = 0.003); being male (OR = 1.95, 95% CI:1.32 to 2.55, *p* = 0.0009) and combined anterior-posterior surgery (OR = 1.77, 95% CI: 1.17 to 2.70, *p* = 0.007). Other risk factors with promising but limited data included nasogastric tube presence.

**Conclusions:**

There is very low certainty evidence that the risk factor groups of tracheostomy, male gender and combined anterior-posterior neck surgery can predict dysphagia in cSCI. More robust and large-scale research with standardised outcome measures is required to explore the independent prognostic factors of dysphagia in cSCI.

## Introduction

Cervical Spinal Cord Injuries (cSCI) are defined as damage to the cervical portion of the spinal cord (C1-C8). They can either be caused by traumatic injury e.g. sporting injuries, road traffic accidents or non-traumatic injuries e.g. tumours, spinal infarctions [1]. Classification is completed by the American Spinal Injury Association (ASIA) (2021) impairment scale which is used to categorise the severity of the SCI both by neurological level and degree of completeness. It infers the level of damage by assessing the degree of motor and sensory loss. The higher the cSCI, the more extensive the motor, sensory and autonomic impairments. High cSCI (C1-C4) have the greatest impairments due to disruption in the corticospinal tracts resulting in tetraplegia (loss of movement of all four limbs), impaired sensation and respiratory dysfunction due to diaphragmatic paralysis, requiring the need for mechanical ventilation [2].

Individuals with cSCI demonstrate a uniquely high prevalence of swallowing impairment compared with other injury levels [3, 4]. The incidence of dysphagia in SCI has been shown to vary dramatically between 16–80% [5]. This variation could partly be explained by the reliance to assess dysphagia using bedside swallow assessment (BSE) which may miss clinical features e.g., silent aspiration. Whereas instrumental assessments, Videofluoroscopy (VFSS) and/or Fibreoptic Endoscopic Evaluation of Swallowing (FEES), may detect more clinical features. There is also heterogeneity in the outcome measures used to measure dysphagia in clinical practice and research e.g. Penetration-Aspiration Scale (PAS) [6], Functional Oral Intake Scale (FOIS) [7]. Accurate identification of prevalence and occurrence of dysphagia in cSCI may improve awareness of the condition and support timely assessment. Earlier identification and management are paramount to ensuring a reduction in complications such as chest infections and aspiration pneumonia.

Dysphagia in cSCI remains under-researched relative to its clinical importance highlighting the need for further investigation into mechanisms involved as this would inform targeted management and rehabilitation plans [8]. The potential risk factors for dysphagia in cSCI are poorly understood, although a wide range of factors have been explored in previous research such as injury-related e.g. neurological level of injury [9, 10], baseline characteristics of patients e.g. age and gender [9, 11], and intervention-related factors e.g. cervical fixation surgery [12]. The evidence base on these potential risk factors is all either outdated or suggests conflicting results as discussed in the paragraph below.

Only one earlier systematic review investigating risk factors for dysphagia in cSCI was identified [9]. The review included data from up to five studies, highlighting the need for a more up-to-date review with a larger evidence base. Neurological level of cSCI was identified as a risk factor but only one [cohort] study was included and therefore a metanalysis could not be completed [9]. Further research on whether neurological level influences dysphagia in cSCI provides mixed results [10, 11]. A more established risk factor for dysphagia is complete cSCI (AIS A) showing substantially higher rates for both temporary and permanent dysphagia in comparison with incomplete injuries (AIS B-D) [13]. However, no studies investigate the combined AIS score of both neurological level and level of completeness e.g. C2 AIS A versus C2 AIS D. In terms of further potential risk factors there are those associated with the clinical interventions for managing the cSCI, such as neck surgery [12, 14], having a tracheostomy in situ and need for ventilator assisted breathing [15, 16].

Among baseline characteristics of people affected by cSCI, older age (>60–65 years) has been consistently shown to be a significant risk factor for developing dysphagia even when adjusting for other confounding variables [10, 17]. Hypotheses have been made linking aging to increased risk of sarcopenia and reduced cough strength, as effective cough strength is closely linked to swallowing safety, as it assists with airway clearance following aspiration [18]. Current evidence demonstrates inconsistent findings regarding gender as a risk factor for dysphagia in cSCI [10, 16].

Identifying the risks for dysphagia in cSCI can be further complicated by clinical pathway variation, pre-existing medical conditions, the impact of deconditioning, differences in treatment locations/practices, treatment pattern differences as well as how dysphagia is assessed [18–20].

To address the evidence uncertainties identified above, this review is to critically appraise and summarise UpToDate research evidence on the risk factors for developing dysphagia in cSCI. The importance of completing this review is to ascertain risk factors to guide dysphagia assessment and management which can subsequently reduce secondary complications such as chest infections and aspiration pneumonia. This systematic review links to The James Lind Alliance Top 10 Research Priorities for SCI [21] which highlight the importance of prevention and management of complications, respiratory complications and specialist rehabilitation services which dysphagia could contribute towards, although not specifically mentioned.

## Methods

The review protocol was formally registered in the International Prospective Register of Systematic Reviews (PROSPERO) (Registration No. CRD42025630835) on 15^th^ January 2025. This protocol followed the guidance of Preferred Reporting Items for Systematic Reviews and Meta-analyses (PRISMA-P) [22].

## Inclusion and exclusion criteria

The inclusion and exclusion criteria were developed in relation to the type of study, participants, exposure and outcomes (PEO). Given the review’s focus on identifying risk factors associated with dysphagia following cSCI, the PEO framework was used to formulate the review question, as this is more appropriate for aetiology and prognostic factor reviews than the traditional PICO framework [23]. Randomised Control Trials (RCTs), non-randomised control trials and non-randomised observational studies (prospective and retrospective cohort studies and cross-sectional studies) were included. Case control, case series, case reports and surveys were excluded. Studies in all languages were included.

Studies included those over age 16 years (as children over 16 are often treated in adult areas), with cSCI (C1-C8 as identified by ASIA), traumatic and non-traumatic injury, including concurrent brain injury and having oropharyngeal dysphagia. We included studies with the presence of dysphagia outcome determined by a dysphagia assessment (BSE, VFSS and FEES) using a validated e.g. PAS or non-validated outcome measure e.g. author defined definition.

Exclusion criteria were those below age 16 years, pre-existing oesophageal dysphagia or oropharyngeal dysphagia, any non-cervical SCI (T1 and below). Intervention criteria did not apply. We considered any risk factor eligible e.g. tracheostomy.

## Search strategy

The search strategy was developed using search terms based on key words from a relevant published paper, Hamilton et al. [24], alongside Peer Review of Electronic Search Strategies (PRESS) [25] and PEO [26]. Each PEO concept became a search term for the database with further synonyms derived e.g. Cervical Spinal Cord Injury, tetraplegia. We also used related MeSH terms and considered British and American spelling variations e.g. Fibre vs Fiber. The Boolean operators, “AND”, “NOT”, “NEAR?” and “OR” were used. Further opinion on the search strategy was gained by an experienced local librarian, and it was also reviewed by a peer Speech and Language Therapist (JM). Testing and reiteration of the search terms occurred by running the search, checking if key papers were missing and reviewing the search terms and modifying accordingly. This occurred until irrelevant papers were reduced and relevant papers were returned with a final search string reached for each database (Supplementary Materials Appendix 1).

The following bibliographic databases were searched, from their inception, on the 16^th^ January 2025: Medline (via Ovid) including In-Process and other Non-indexed Citations, Embase (via Ovid), The Cochrane Central Register of Controlled Trials (CENTRAL; 2025; Issue 1) in the Cochrane Library, Cinahl (via EBSCOhost) and Web of Science Core Collection (Via Clarivate) (1900 to 16th January 2025). A repeat search was completed on 27th July 2025. There were no restrictions on publication year. Scopus was searched for conference abstracts and conduct citation tracking. ProQuest Dissertations and Theses Global was searched to identify unpublished studies. The reference lists of all included studies as well as previous systematic reviews within this topic area were reviewed [9, 27, 28]. Authors of included studies were contacted to determine whether they had unpublished or ongoing relevant work that they were prepared to share. The Clinical trial registries ClinicalTrials.gov and WHO International Clinical Trials Registry Platform (ICTRP) were searched to identify ongoing and unpublished trials.

## Screening and selection

Screening of titles and abstracts, followed by full text screening, was completed by two reviewers independently (SL and IB) using Rayyan Systematic Review Screening Platform [29]. Screening used a pre-determined screening checklist with the inclusion and exclusion criteria at population level, study type, outcome measures such as PAS. Deduplication was completed by Rayyan prior to screening commencing and double checked by a reviewer (SL). Inter-rater agreement was calculated using Cohen’s kappa at title and abstract screening, with disagreements resolved through discussion A third reviewer (CS) resolved conflicts at title and abstract as well as full text screening.

## Data extraction

A data extraction form was developed in Microsoft Excel which was piloted by two reviewers (SL and CS). This included:

- **Study Identifiation Details e.g**., Author, year
- **Participants e.g**., Total sample size, mean age (SD), proportion of male participants, Neurological Level of cSCI, injury completeness,
- **Exposure -** Presence of candidate risk factor (with definition),
- **Outcomes -** Association between risk factors and dysphagia e.g. numbers with/without dysphagia by exposure status, relevant binary/continuous data and effect estimates e.g. ORs with standard errors if reported.

The data was extracted by a reviewer (SL), with a new excel spreadsheet for each study, and with a second reviewer (IB) checking all the data for accuracy of the extraction. The two reviewers (SL and IB) met to resolve any conflicts with data extraction. Study authors were contacted to resolve any further uncertainties with a maximum of three email attempts.

## Critical appraisal and risk of bias

All included studies were critically appraised using a published and validated risk of bias tool, to gain a score on the credibility of each included study. The Newcastle Ottawa Scale (NOS) [30] was the main appraisal tool chosen as most included studies were cohort. The score is out of 9 with 0–3 being low methodological quality, 4–6 medium methodological quality and 7–9 being high. For the one cross-sectional study, the modified NOS was used [31]. This is like the NOS with different signalling questions and is scored out of 10. All quality assessments for individual studies were completed via an excel spreadsheet independently by two reviewers (SL and IB) who met to discuss any discrepancies prior to a final quality rating being given.

## Data analysis and synthesis

Risk factor data were categorised into six predefined key risk factor groups based on conceptual similarity across included studies (e.g., gender). Also, they represent some of the key domains influencing swallowing function e.g. airway modification (tracheostomy), patient-related physiology (age, gender), neurological and iatrogenic factors affecting swallow biomechanics (injury level, completeness, and surgical approach). Other risk factors which could be judged to be clinically relevant were excluded as there was inconsistent reporting across studies / to maintain feasibility and reduce heterogeneity e.g. mechanical ventilation.

Descriptive statistics relating to study characteristics, dysphagia outcome measures and risk factors from individual studies were pooled using Microsoft Excel Formulae and presented in a summary table. Clinical heterogeneity (e.g., differences in participant populations, injury characteristics, and outcome definitions) and methodological heterogeneity (e.g., variation in study design, measurement tools, and risk factor definitions) across studies was assessed. Where appropriate, data were pooled across studies for factors reported in more than one study. As not all studies reported every variable, the total number of participants differed between outcomes, and percentages are presented relative to the number of participants available for each measure. When substantial heterogeneity was present, findings were synthesised narratively, with consideration to the consistency of association between pooled results and studies reporting the same predictors that could not be statistically combined.

Meta-analysis was completed in Review Manager Version 5.4.1 [32] when the study characteristics were similar. For binary data, a Mantel-Haenszel random-effects model was used to account for heterogeneity, with summary univariable odds ratios (ORs) and risk ratios (RRs) calculated along with 95% confidence intervals (CIs). ORs were chosen so multiple regression models could be completed and RRs to compare to a previous systematic review which only used this effect measure [9]. Where multiple regression models were reported, an Inverse Variance method was used and were prioritised over univariate data to account for confounding. For continuous data, Mean Differences (MDs) with 95% CIs were calculated using a generic inverse-variance random-effects model. For meta-analysis, effect estimates for the six main risk factors were tabulated as univariable and multivariable ORs and RRs. We used the χ^2^ test and the Higgins and Thompson I^2^ statistics to quantitatively assess statistical heterogeneity; and we considered that a value of ≥ 75% suggested high heterogeneity. Forest plots were used to visually display pooled estimates for the key risk factors. We did not perform funnel plots to assess the publication bias nor used subgroup analysis to explore the cause of heterogeneity as no analysis included more than ten studies. However, we did perform a sensitivity analysis in which we excluded studies of low quality to assess the robustness of the related primary analysis.

## Certainty of evidence

The strength of the body of evidence was assessed by Grading of Recommendations Assessment, Development and Evaluation (GRADE) [33]. The quality of evidence was assessed across the following domains: **Risk of bias,** **Inconsistency,** **Indirectness,** **Imprecision** and **Publication bias**. Quality was determined as high, medium, low or very low by a reviewer (SL). GRADE was applied at the level of individual risk factors, as recommended, to evaluate the overall certainty of evidence for each exposure–outcome relationship across studies, rather than at the level of individual studies, which is addressed through risk of bias assessment. In addition, the 6 risk factors chosen had enough studies to combine to make the GRADE analysis feasible.

All studies started with a low GRADE score as they were all observational and could be downgraded by one or two points by assessing the above domains. In rare cases, upgrading can occur where there is certainty of large effects e.g., a confidence interval between 3 and 5, as the results are unlikely to be due to bias. Upgrading can only occur by two levels.

## Results

### Study selection process

**Fig. 1:**
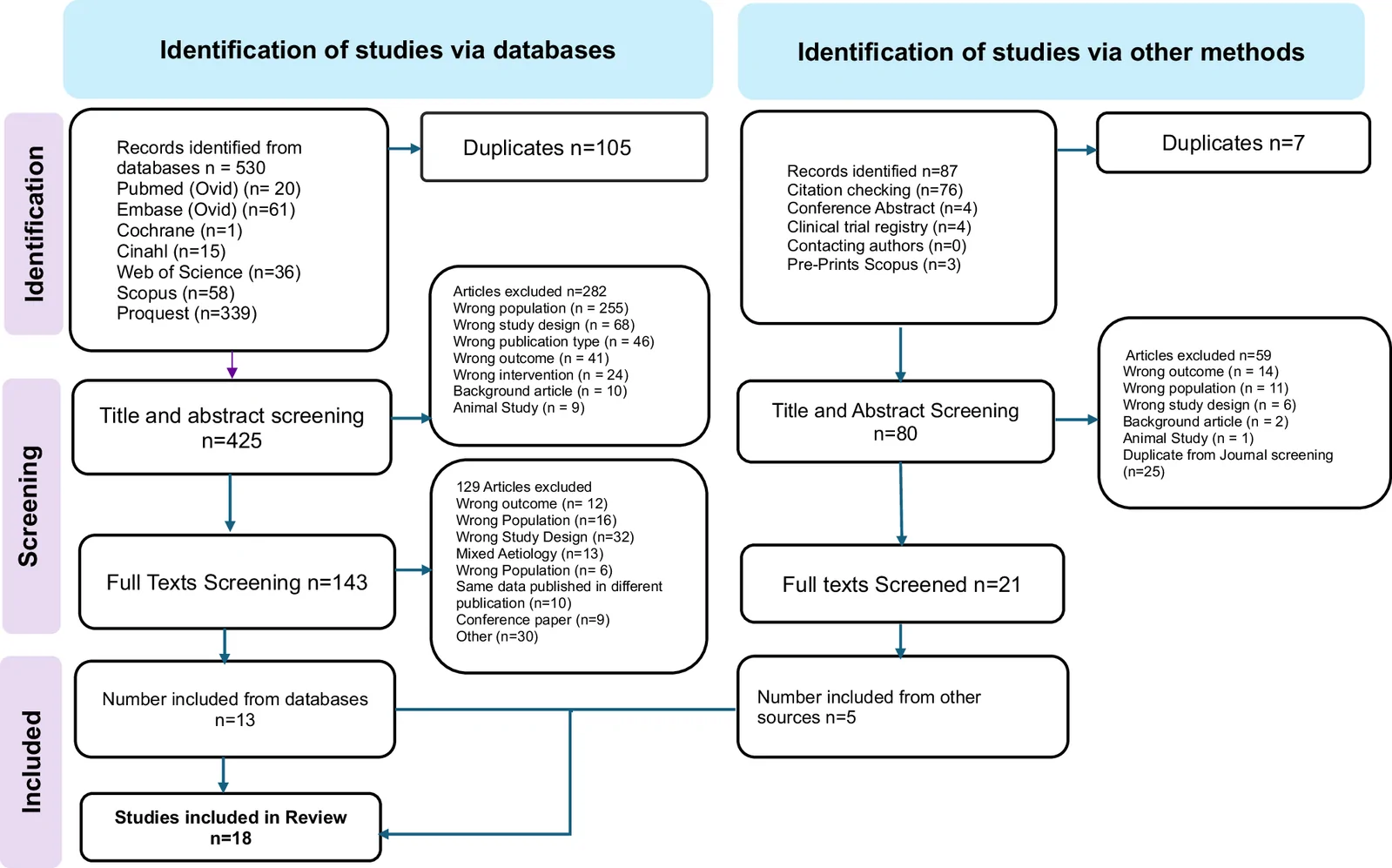
Adapted PRISMA flow diagram from Rayyan [27] detailing the search and study selection process, including records identified through database searches and other sources, screening, eligibility assessment and final inclusion in the review.

The study screening and selection process is shown in the modified PRISMA diagram developed using Rayyan [29] (Fig. 1). Electronic databases returned 530 titles and abstracts. Following removal of 105 duplicates via Rayyan, 425 articles from databases were screened at title and abstract. At this stage, 282 were excluded with the most common reason being that they examined populations outside the scope of this review. Through identification of studies via other methods, 80 articles were screened at title and abstract with the removal of 7 duplicates via Rayyan. Fifty-nine articles were removed at this stage, with the most common reason being duplication with studies retrieved from databases.

At full-text screening there were 143 articles with 129 excluded at this stage mainly for being the wrong study design. From studies identified from other methods, 21 studies were carried forward to full-text screening, with 16 excluded at this stage, the main reason being the wrong study aim. In total, 18 studies were included in this review, 13 from database searching and 5 via other sources fulfilling the eligibility criteria (Supplementary Materials Appendix 2 for table of included studies characteristics and Appendix 3 for references of included studies). Inter-rater agreement for title and abstract screening was substantial (κ = 0.68).

### Descriptive statistics

The higher-level descriptive statistics of the 18 included studies are shown in Table 1 and Supplementary Materials Appendix 4 are described below:

- **Study Characteristics**: The total sample size across the included studies was 2,258 participants. Individual study sample sizes varied with a median 99 participants (IQR: 70–146). Most studies were retrospective cohort (n = 12, 66.7%), followed by prospective cohort (n = 5, 27.8%) and a single cross-sectional study (n = 1, 5.6%). Only 22.2% (n = 4) of studies reported follow-up data, with a mean follow-up of 104.5 days across studies. Research teams from Korea and the USA both contributed the most studies (n = 5 each, 27.8%), followed by Japan (n = 4,22.2%), Germany 11.1% (n = 2) with Finland and New Zealand each contributing the least (n = 1, 5.6%).
- **Outcome measures**: The use of outcome measures varied, with several studies reporting more than one outcome, resulting in a total of 21 outcome measures across the 18 included studies. PAS was the most frequently used validated outcome (n = 4, 19.0%), followed by the FOIS (n = 2, 9.5%). Other validated measures, including the Food Intake Limit Scale (FILS), Yale Pharyngeal Residue Severity Rating Scale, Dysphagia Severity Scale (DSS), and Videofluoroscopic Dysphagia Scale (VDS), were each used once (n = 1, 4.7%). Notably, 38.1% (n = 8) of reported outcomes were non-validated, such as categorisation by parenteral nutrition versus oral intake. Additionally, 9.5% (n = 2) of studies did not report how dysphagia was measured.
- **Risk factors:** Because data were pooled from multiple studies with variable outcome reporting, the total number of participants differed between analyses; consequently, absolute counts and percentages may not align across outcomes. Participants with dysphagia had a ***higher mean age*** than those without dysphagia (57.74 ± 28.32 vs. 50.61 ± 22.02 years) and were more likely to have ***high-level (C1–C4) injuries*** (207 participants, 33.2% vs. 85 participants, 17.6%) and ***complete injuries (AIS A)*** (152 participants, 34.1% vs. 203 participants, 30.3%). Markedly higher rates of ***tracheostomy*** (241 participants, 47.9% vs. 229 participants, 18.7%) and ***nasogastric tube*** use (67 participants, 67.0% vs. 21 participants, 27.6%) were also observed in the dysphagia group.

**Table 1:** Higher-level descriptive statistics of the included studies.

Items	Summary Statistic
**Study Design (n** = **18)**	
Retrospective Cohort Studies n (%)	12 (66.7%)
Prospective Cohort Studies n (%)	5 (27.8%)
Cross Sectional Studies n(%)	1 (5.6%)
**Location n (%) (n** = **18)**	
Korea	5 (27.8%)
Japan	4 (22.2%)
Finland	1 (5.6%)
USA	5 (27.8%)
Germany	2 (11.1%)
New Zealand	1 (5.6%)
Sample Size (Median) n = 2258	99 (70 – 146)
**Outcome measure used n (%)** (n = 22 – some studies used more than one outcome measure)	
PAS	4 (21.7%)
FOIS	2 (9.5%)
FILS	1 (4.7%)
Yale	1 (4.7%)
DSS	1 (4.7%)
VDS	1 (4.7%)
Quality of Life	0 (0%)
Non-validated	8 (38.1%)
Not recorded	2 (9.5%)
**Follow up**	
Yes	4 (22.2%)
No	14 (88.8%)

*PAS* Penetration Aspiration Score, *FOIS* Functional Oral Intake Scale, *FILS* Food Intake Limit Scale, Yale Pharyngeal Residue Severity Rating Scale, *DSS* Dysphagia Severity Scale (DSS), *VDS* Videofluoroscopic Dysphagia Scale.

Participants with dysphagia had ***lower mean percentage predicted forced vital capacity*** (44.29 ± 19.1 vs. 59.98 ± 16.83) and ***higher rates of mechanical ventilation*** (58 participants, 53.7% vs. 30 participants, 44.1%). ***Cervical neck surgical interventions*** were more frequent among those with dysphagia, particularly posterior approaches (121 participants, 34.4% vs. 213 participants, 25.4%) and combined anterior–posterior procedures (45 participants, 32.1% vs. 157 participants, 22.7%).

Other prevalent risk factors among participants with dysphagia included ***concurrent brain injury*** (61.7% vs. 39.5%), ***cervical collar use*** (66.7% vs. 29.5%), ***halo fixation*** (32.5% vs. 7.7%), ***history of bronchoscopy*** (68.5% vs. 33.0%), and ***pneumonia*** (48.4% vs. 10.2%). Overall, dysphagia was more frequently observed in patients with greater injury severity, respiratory compromise, and additional neurological or procedural complications.

### Results of risk of bias and applicability

The table of the results for the completed NOS can be found in Supplementary Materials Appendix 5. Of the 17 cohort studies, five studies [S2, S6, S8, S9, S11] were rated as high quality, primarily due to strong cohort representativeness, clear exposure ascertainment and the consistency of how dysphagia was measured. The rest of the 12 cohort studies [S1, S3, S4, S5, S6, S10, S12, S13, S14, S15, S16, S17] were rated as moderate quality often due to lack of adjustment for key confounders and incomplete follow-up.

We rated Yoon et al. [S18], the only cross-sectional study included in this review, as moderate quality due to lack of sample size justification, no information on non-respondents and not all confounders controlled for.

### Meta-analysis

There were 15 risk factors identified overall with the meta-analysis results given in (Supplementary Materials Appendix 6). The univariate and multi-variate ORs are given for the six main risk factors in Table 2.

**Table 2:** Results for all odds ratios calculated in the univariate and multivariate meta-analysis for the 6 most clinically relevant risk factors.

Risk Factor	Univariate OR (95% CI) p-value	Multivariate Logistic Regression OR (95% CI) p-value
Tracheostomy (Yes vs No)	N = 8 [S1, S6, S7, S10, S11, S15, S16, S17] (780 participants) 3.52 (1.57 to 7.85) *p* = 0.002	N = 5 [S3, S5, S8, S9, S11] (1,028 participants) 4.91 (1.74 to 13.86) *p* = 0.003
Combined Surgical Approach (Yes vs No)	N = 5 [S1, S9, S12, S13, S16] (880 participants) 1.77 (1.17 to 2.70) *p* = 0.007	Not Applicable (Univariate data only)
Neurological Level (High vs Low)	N = 4 [S6, S15, S17, S18] (229 participants) 1.38 (0.73 to 2.59) *p* = 0.32	N = 3 [S5, S8, S9] (850 Participants) 1.31 (0.55 to 3.11) *p* = 0.54
Completeness of cSCI (Complete vs Incomplete)	N = 6 [S3, S9, S15, S16, S17, S18] (815 participants) 1.42 (0.79 to 2.55) *p* = 0.24	N = 2 [S6, S8] (186 participants) 1.07 (0.16 to 7.09) *p* = 0.94
Gender (Male vs Female)	N = 9 [S3, S5, S6, S8, S9, S10, S11, S15, S17] (1, 295 participants) 1.95 (1.32 to 2.88) *p* = 0.0009	N = 1 [S8] (149 participants) 1.08 (1.04 to 1.12) *p* < 0.0001
Age (continuous)	Calculated from Mean Difference (see Supplementary Appendix 5)	N = 3 [S6, S8, S9] (589 participants) OR = 1.04, CI 95% 1.00–1.08, *p* = 0.07

N = number of studies included in the meta-analysis.

**Fig. 2:**
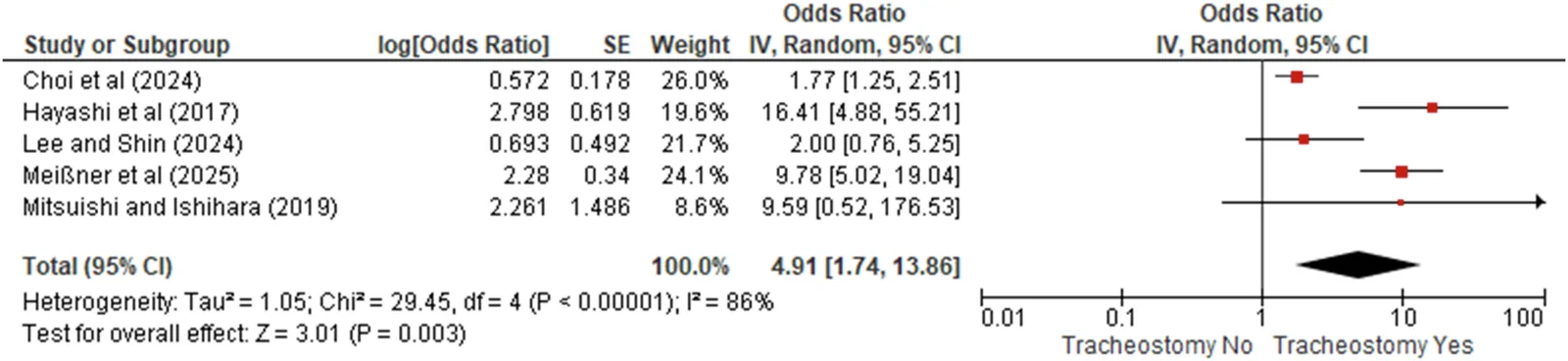
Forest plot of multivariate logistic regression results examining tracheostomy as a risk factor for dysphagia. The figure displays log ratios (OR), standard errors (SE), individual study odds ratios (Ors) with 95% confidence intervals (CIs) and the pooled random-effects estimate.

Three risk factors were statistically significant from combining multiple studies: Tracheostomy, male gender and anterior-posterior combined neck surgery. Across five studies [S3, S5, S8, S9, S11] (1,028 participants), tracheostomy was consistently associated with increased odds of dysphagia when examined with multivariate logistic regression (Fig. 2). The pooled analysis demonstrated nearly a fivefold higher risk (OR = 4.91, 95% CI: 1.74 to 13.86), *p* = 0.003) for patients with a tracheostomy compared to those without. Although effect sizes varied substantially between studies (I^2^ = 86%), the overall association remained statistically significant.

Nine Studies [S3, S5, S6, S8, S9, S10, S11, S15, S17] (1,295 participants) reported being of male gender as a risk factor for dysphagia via univariate results. Being male demonstrated a consistent and significant associated risk factor for dysphagia with a pooled OR of 1.95 (95% CI: 1.32 to 2.88, *p* = 0.0009). This suggests that males have an approximately 1.95 greater odds of developing dysphagia compared to females. The confidence interval was narrow and the effect sizes consistent (I^2^ = 0%). Only one study, Lee and Shin [S8], reported a logistic regression result, therefore only univariate results could be used in the meta-analysis, therefore confounding could not be controlled for.

**Fig. 3:**
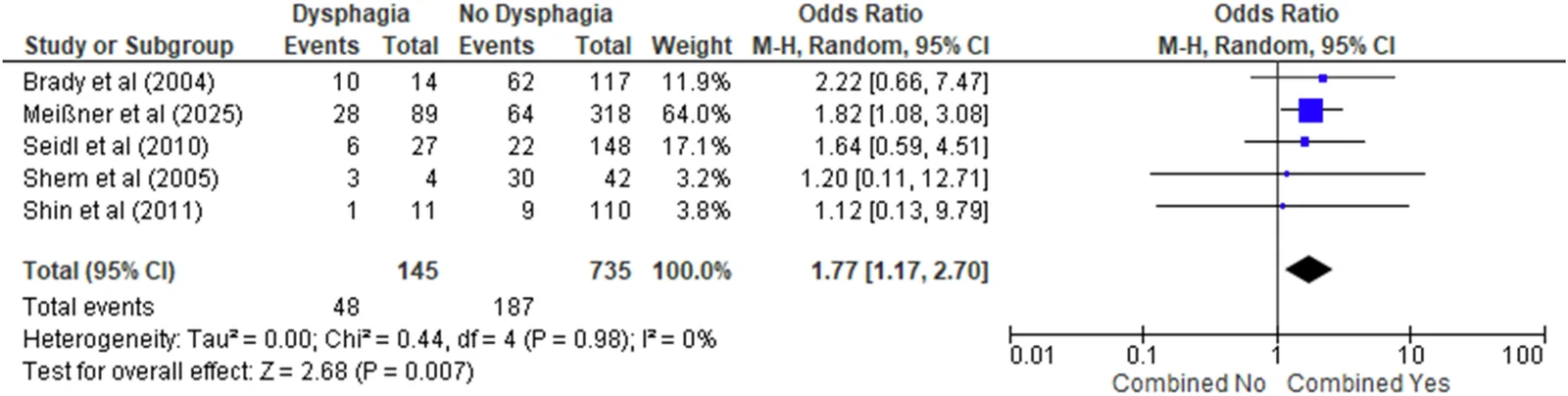
Forest plot showing the univariate association between combined anterior-posterior surgery and dysphagia. The figure includes study-level dysphagia event counts, total participants, individual odds ratios (ORs) with 95% confidence intervals (CIs) and the pooled random-effects model estimate.

Five studies [S1, S9, S12, S13, S16] (880 participants) reported on a combined anterior and posterior surgical approach as a risk factor. When completing the meta-analysis with univariate data a combined surgical approach was significantly associated with an increased risk of dysphagia (OR = 1.77, 95% CI: 1.17 to 2.70, *p* = 0.007) with low heterogeneity (I^2^ = 0%) (Fig. 3). Only a univariate analysis could be undertaken with these data.

Other significant risk factors include presence of nasogastric tube (NGT) (OR = 4.99, 95% CI = 1.20 to 20.73, *p* = 0.03 [S15, S17], undergoing a bronchoscopy (OR = 3.87, 95% CI = 1.06 to 14.14, *p* = 0.04) [S6, S15] and history of pneumonia (OR = 5.21, 95% CI = 1.89 to14.32, *p* = 0.001) [S15, S16]. However, as these results were gained from only two studies combined via univariate analysis, the results are exploratory rather than definitive.

### GRADE assessment results

The GRADE ratings were completed for the 6 main risk factors (Supplementary Materials Appendix 5). All but one of the main 6 main risk factors were rated as very low quality, other than gender rated as low. This means that substantial caution should be taken when interpreting the results overall.

### Sensitivity analysis excluding studies of low quality

Six lower-quality studies [S7, S10, S12, S13, S16, S17] (NOS rating 4-5/9) were removed for this sensitivity analysis. The pooled OR rose from 3.52 (95% CI = 1.57 to 7.85, *p* = 0.002) to 5.73 (95% CI = 2.59 to 12.65, *p* < 0.0001) strengthening the tracheostomy-dysphagia association. However, wider confidence intervals indicate reduced precision, likely due to only four studies versus eight in the main analysis, increasing the influence of individual studies. There were no low-quality studies in the multivariate analysis, therefore there must be another source of heterogeneity.

## Discussion

Having a tracheostomy, being male and combined anterior-posterior surgery were the most significant risk factors associated with dysphagia for people with cSCI. Overall, the quality of the evidence returned was low to very low via GRADE due to variation in the outcome measures used, as well as reduced adjustment for confounding variables suggesting that substantial caution should be applied when interpreting the results. Thus, it is uncertain whether these factors are prognostic for having dysphagia post cSCI.

The results suggest that having a tracheostomy is a strong predictor of dysphagia in cSCI with multivariate results. This has clinical significance as tracheostomy may impair the swallow mechanism. A cuffed tracheostomy has been associated with reduced hyolaryngeal elevation, likely due to mechanical tethering and altered subglottic pressure but studies have shown there be no change in hyolaryngeal elevation pre- and post- decannulation as well as patient heterogeneity [34, 35]. Disuse atrophy of swallowing musculature may occur secondary to reduced airflow and altered oropharyngeal use in particularly in cuffed tracheostomised patients [36, 37]. Tracheostomy can reduce airflow through the upper airway, diminishing pharyngeal and laryngeal sensation and impairing swallow initiation and airway protection [38, 39]. While causality remains debated, the physiological rationale and clinical association justify its inclusion of tracheostomy as a key risk factor and strengthening the need for through dysphagia assessment in this client group.

Male gender was identified as another clinically significant risk factor. However, this may not represent a true biological risk, but rather a confounding effect; males are disproportionately represented among individuals with cSCI due to higher injury rates [40]. This could explain the greater frequency of dysphagia observed in men. Therefore, clinical focus should shift to other risk factors.

Combined Anterior-Posterior surgery was found to be a statistically significant risk factor when compared to either surgical approach in isolation. This significance emerges because the subgroups consistently carry higher risk which becomes more pronounced when not diluted by lower risk surgery subgroups. Dysphagia may arise from both the multifactorial mechanical and neurological factors that can be affected by the anterior-posterior surgery. The duration of the surgery is likely to be longer with prolonged anterior retraction and multilevel exposure. This can lead to oedema and prevertebral swelling, but it is worth noting that the impacts of these surgical side effects are more likely to resolve over time [41]. Multiple surgeries may be resulting in prolonged and recurrent airway intubation which may be a confounding variable for why combined surgery increases the risk of dysphagia.

Age, neurological level and level of completeness, despite showing results in the expected direction, did not reach statistical significance, likely reflecting limited power or heterogeneity between studies. Clinically, these risk factors should not be disregarded as they remain plausible contributors to outcome risk based on biological mechanisms and prior research. Thus, careful consideration of these variables in patient assessment and management is still warranted but further research is needed to clarify their roles. In a recent retrospective cohort study, it was shown that there was increased risk of pharyngeal residue in those with cSCI and increasing age [42]. However, this research only uses VFSS, highlighting the need for further research linking risk factors and physiological characteristics with FEES as some physiological characteristics may be missed e.g. presence and location of secretions, vocal cord adduction, laryngeal pathology, pharyngeal and laryngeal sensation [43, 44].

Emerging risk factors including presence of nasogastric feeding tube, having undergone a bronchoscopy, and history of pneumonia showed some trends to potentially causing dysphagia, but further research is needed as limited studies were combined for meta-analysis. It could suggest that invasive airway or feeding interventions exacerbate swallowing impairment with hypotheses potentially being mucosal irritation [45].

### Limitations of this review

In most studies, the time post injury was not recorded. Swallowing difficulties are likely to be worse immediately post injury with a level of recovery expected with time and healing. Without clear recording of when assessments of dysphagia are completed, it is impossible to know whether these reflect acute, subacute or chronic stages as well as spontaneous recovery, limiting the understanding of when dysphagia is most problematic and the true extent of dysphagic characteristics.

This systematic review was limited to high cSCI. However, there are studies that show dysphagia can occur at lower SCI levels. For example, Valenzano et al. [19] included two thoracic level SCI patients with swallow parameters showing atypical values. This could be due to those with an injury level at the 6th thoracic segment or higher having evidence of respiratory complications and autonomic dysregulation affecting swallow-respiratory cycles [46]. Therefore, further research to establish clinical characteristics of all SCI levels may be required to gain a full picture.

Five studies were deemed to be of lower quality mainly due to confounding variables not being controlled for in statistical analyses. This could inflate the overall results of this meta-analysis. There were multiple aspects across all the studies included where there were concerns about heterogeneity, confirmed by the I^2^ results in the meta-analyses. The main ones were how dysphagia was measured e.g. some studies measured the presence or absence of dysphagia without a validated tool. Some results were reported as an ordinal scale e.g. individual PAS scores, whilst others were reported in binary form e.g. PAS < 2. This resulted in difficulties combining the results. Using binary data also reduced the available information e.g. PAS score of 3 and 8 both are rated in the same category in PAS < 2 but have different clinical implications. This can result in reduced statistical power as it reduces variability with failures to pick up associations. The meta-analyses resulting from univariate data should be interpreted with caution as dichotomisation of dysphagia outcomes is likely to result in misclassification bias (as well as contributing to reduced precision and obscuring severity). In contrast, multivariate meta-analyses which retain ordinal or continuous data, as well as adjusting for confounders, are likely to provide more accurate estimates despite the reduced number of pooled studies.

The outcome measures used to quantify dysphagia varied between studies resulting in heterogeneity and could be an argument for not completing a meta-analysis e.g., one study used an indirect marker of whether the person was on full oral intake or parental nutrition upon discharge as a marker for dysphagia [S17]. This indirect measure may miss aspects of dysphagia e.g. people can still manage regular oral intake and still have pharyngeal pooling. It was noted a large proportion of studies defined dysphagia exclusively using penetration-aspiration measures. While PAS is a validated and very clinically relevant outcome measure for quantifying airway invasion, it only captures one symptom of a swallowing difficulties, ignoring physiological deficits e.g. reduced pharyngeal stripping wave. The presence of dysphagia may be underreported due to this narrow definition and introduce measurement bias to this review.

Variation in how risk factors were defined may have introduced further indirectness; for example, the presence of a tracheostomy may have referred either to its presence at the time of swallowing assessment or to any history of tracheostomy during admission. Kirshblum et al. [16] separated out the data for history of tracheostomy and presence. during rehabilitation/time of VFSS, but it is not clear with other studies whether these were counted together or separately. Therefore, the combined effect sizes may not reflect a single underlying effect. Alternative definitions (history/ever) could have been used in a further sensitivity analysis.

A source of bias is that the GRADE assessment was only completed for the 6 main risk factors and only by a single reviewer (SL) secondary to time constraints. Other risk factors could be determined to be just as clinically relevant, but the use of GRADE was not appropriate due to the lack of studies in these areas e.g. mechanical ventilation. There was also lack of RCTs in this research resulting in overall the GRADE assessment being low. However, it would be impossible and unethical to complete RCTs into risk factors e.g. assigning patients to a surgical route linked to higher aspiration risk.

### Comparison to previous systematic review

Iruthayarajah et al. [9] included 11 papers (1, 227 participants) in their systematic review of dysphagia risk factors in cSCI compared to 18 papers (2, 245 participants) in this review. They identified seven risk factors including age and injury severity. The current review found over 15 risk factors, with six supported by multiple studies with adjusted analyses used which Iruthayarajah et al. [9] did not. Iruthayarajah et al. [9] only reported on two risk factors from multiple studies: tracheostomy and cervical surgery. When directly comparing the two systematic reviews univariate analyses, the tracheostomy risk ratio (RR) fell from 3.67 (95% CI = 1.82 to 7.42), *p* < 0.0001(5 studies, 805 participants) to 2.10 (95% CI = 1.29 to 3.44) *p* = 0.003 (8 studies, 780 participants), reflecting weaker but still significant evidence for the risk with more studies combined, albeit less participants. Cervical surgery had a RR = 1.30 (95% CI = 1.09 to 1.55), *p* = 0.0036 (2 Studies, 318) which reduced to a RR = 1.15 (95% CI 0.74 to 1.80) *p* = 0.53 (9 studies, 1,196 participants) for this systematic review, showing that with a larger sample size statistical significance was not achieved. Notably, they reported RR rather than odds ratio (OR) which may better qualify event likelihood. However, RR cannot be calculated from multiple logistic regression, so direct comparison was not always possible. Thus, comparisons across reviews using different analytic approaches should be interpreted with caution, though this current review is likely to be more accurate as it includes meta-analysis from logistic regression.

## Conclusion

The complexity of determining clinical characteristics of dysphagia within cSCI is further complicated by the many potential risk factors associated with it. Key risk factors are the presence of a tracheostomy and combined anterior-posterior surgery with emerging risk factors such as NGT needing further research to strengthen the association. The certainty of the evidence is very low warranting caution in interpreting the results, suggesting hypothesis generation rather than changing policy. Future studies are needed with standardised methodologies, consistent validated outcomes and multivariate analyses to clarify these relationships and inform future clinical policy and guidelines.

## Supplementary information

Supplemental Material

## Data Availability

Additional data can be requested via Sharon.leigh@nhs.net.
