# Supplementary material for: Risk Factors associated with dysphagia for people with Cervical Spinal Cord Injury (cSCI): A Systematic review and Meta-analysis
Source: Spinal Cord. 2026 Aug 26;64(10):865–73. doi: 10.1038/s41393-026-01258-2 (PMC13668727; doi:10.1038/s41393-026-01258-2)
Supplement: Supplementary file 1 [file 41393_2026_1258_MOESM1_ESM.docx]

**Supplementary Materials**

**Appendix 1 Summary of the search strategy applied across all databases, including Medline, Embace, CINAHL, Cochrane Library, Web of Science and ProQuest Dissertations and Theses. Search strategies combined terms related to spinal cord injury, dysphagia and clinical assessment of swallowing.**

| **Database** | **Search Strategy** |
| --- | --- |
| Medline and Embase (Via Ovid) | 1.Spinal cord injur*.kw. 2. Spinal cord injur*.ab,ti.3. exp Spinal Cord Injuries/ 4. Tetraplegia.kw. 5. Exp Quadriplegia/ 6. Tetraplegia.ti,ab. 7. Quadr?plegia.ti,ab. 8. Quadraplegia.mp.9. Quadraplegia.kw. 10. Quadriplegia.kw. 11. 1 or 2 or 3 or 4 or 5 or 6 or 7 or 8 or 9 or 10. 12. Dysphagia.kw. 13. exp Deglutition Disorders/ 14. Dysphagia.ti,ab. 15. Deglutition disorders.kw. 16. Deglutition disorders.ti,ab. 17. exp Deglutition/ 18. Deglutition.kw. 19. Deglutition.ti,ab. 20. Swallow* disorders.ti,ab,kw. 21. Oropharyngeal dysphagia.ti,ab,kf. 22. 12 or 13 or 14 or 15 or 16 or 17 or 18 or 19 or 20 or 21. 23. Instrumental assessment.ti,ab,kw. 24. Videofluoroscopy.ti,ab,kw. 25. Videofluoroscopic swallow study.ti,ab,kw. 26. Videoswallow.ti,ab,kw. 27. VFSS.ti,ab,kw. 28. VFS.ti,ab,kw. 29. Modified barium swallow study.ti,ab,kw. 30. MBS study.ti,ab,kw. 31. MBSimp.ti,ab,kw. 32. exp cineradiography/. 33. 23 or 24 or 25 or 26 or 27 or 28 or 29 or 30 or 31 or 32. 34. Fib* endoscopic evaluation.ti,ab,kw. 35. (Flexible endoscopic Evaluation adj3 Swallow).ti,ab,kw. 36. FEES.ti,ab,kw. 37. 34 or 35 or 36. 38.Bedside assessment.ti,ab,kw. 39. Bedside swallow evaluation.ti,ab,kw. 40. clinical swallow evaluation.ti,ab,kw. 41. 38 or 39 or 40. 42. 33 or 37 or 41. 43. 11 and 22 and 42 |
| Cochrane library | 1. MeSH descriptor: [Spinal Cord Injuries] explode all trees 2. MeSH descriptor: [Deglutition Disorders] explode all tree 3. Instrumental assessment of swallow 4. #1 AND #2 AND #3 5. (spinal cord injury):ti,ab,kw AND (dysphagia):ti,ab,kw AND (instrumental assessment):ti,ab,kw |
| Cinahl (EBSCOhost) | S1 TI ( spinal cord injury or sci or paraplegic or quadriplegic ) OR AB ( spinal cord injury or sci or paraplegic or quadriplegic ) OR MW ( spinal cord injury or sci or paraplegic or quadriplegic ) OR MH ( spinal cord injury or sci or paraplegic or quadriplegic )  S2 TI ( oropharyngeal dysphagia or swallowing disorders or deglutition disorders ) OR AB ( oropharyngeal dysphagia or swallowing disorders or deglutition disorders ) OR MW ( oropharyngeal dysphagia or swallowing disorders or deglutition disorders ) OR MH ( oropharyngeal dysphagia or swallowing disorders or deglutition disorders )  S3 TI ( videofluoroscopic swallow study or VFS or VFSS ) OR AB ( Videofluoroscopy swallow study or VFS or VFSS ) OR MW ( Videofluoroscopy swallow study or VFS or VFSS ) OR MH ( Videofluoroscopy swallow study or VFS or VFSS )  S4 TI instrumental assessment OR AB instrumental assessment OR MW instrumental assessment OR MH instrumental assessment  S5 TI ( fiberoptic endoscopic evaluation of swallowing or fees ) OR AB ( fiberoptic endoscopic evaluation of swallowing or fees ) OR MW ( fiberoptic endoscopic evaluation of swallowing or fees ) OR MH ( fiberoptic endoscopic evaluation of swallowing or fees )  S6 TI ( fibreoptic endoscopic evaluation of swallowing or fees ) OR AB ( fibreoptic endoscopic evaluation of swallowing or fees ) OR MW ( fibreoptic endoscopic evaluation of swallowing or fees ) OR MH ( fibreoptic endoscopic evaluation of swallowing or fees )  S7 TI ( modified barium swallow study or mbs ) OR AB ( modified barium swallow study or mbs ) OR MW ( modified barium swallow study or mbs ) OR MH ( modified barium swallow or mbs )  S8 TI ( bedside swallow evaluation or clinical swallow evaluation ) OR AB ( bedside swallow evaluation or clinical swallow evaluation ) OR MW ( bedside swallow evaluation or clinical swallow evaluation ) OR MH ( bedside swallow evaluation or clinical swallow evaluation )  S9 S3 OR S4 OR S5 OR S6 OR S7 OR S8  S10 S1 AND S2 AND S9 |
| Web of Science | 1. Bedside assessment (all fields) 2. Bedside swallow evaluation (All fields) 3. clinical swallow evaluation (All fields) 4. FEES (all Fields) 5. **Flexible endoscopic evaluation of swallow** (All Fields) 6. **Fiberoptic endoscopic evaluation of swallow** (All Fields) 7. **Fibreoptic endoscopic evaluation of swallow** (All Fields) 8. **MBSIMP** (All Fields) 9. **modified barium swallow stud*** (All Fields) 10. **modified barium swallow study** (All Fields) 11. **modified barium swallow** (All Fields) 12. **VFSS** (All Fields) 13. **VFS** (All Fields) 14. **videofluoroscopic swallow study**  (All Fields) 15. **video swallow** (All Fields) 16. **videofluoroscopy** (All Fields) 17. **#** **1 OR #2 OR #3 OR #4 OR #5 OR #6 OR #7 OR #8 OR #9 OR #10 OR #11 OR #12 OR #13 OR #14 OR #25 OR #16** 18. **swallowing disorders** (All Fields) 19. **oropharyngeal dysphagia** (All Fields) 20. **deglutition disorders**  (All Fields) 21. **Dysphagia** (All Fields) 22. **#18 OR #19 OR #20 OR #21** 23. **Quadriplegia (All Fields)** 24. **tetraplegia** (All Fields) 25. **spinal cord injury** (All Fields) 26. **#23 OR #24 OR #25** 27. **#17 AND #22 AND #26** |
| ProQuest dissertations and thesis global | (("spinal cord injury" OR tetraplegia OR quadraplegia) AND (dysphagia OR (deglutition disorders) OR (oropharyngeal dysphagia) OR (swallowing disorders)) AND (videofluoroscopy OR (video swallow) OR (videofluoroscopic swallow study) OR VFS OR vfss OR (modified barium swallow) OR (modified barium swallow study) OR MBSIMP) AND ((Fibreoptic endoscopic evaluation of swallow) OR (Fiberoptic endoscopic evaluation of swallow) OR (Flexible endoscopic evaluation of swallow) OR FEES) AND ((Bedside assessment) OR (Bedside swallow evaluation) OR (clinical swallow evaluation))) AND stype.exact(("Scholarly Journals" OR "Dissertations & Theses" OR "Trade Journals" OR "Reports" OR "Wire Feeds") NOT "Books") |

**Appendix 2 Table of Included Studies**

| **Study ID** | **Study Design** | **Location** | **No of Participants** | **Outcome measures with instrumentation used to define dysphagia** | **Time post injury of when dysphagia assessment completed** | **Follow up** |
| --- | --- | --- | --- | --- | --- | --- |
| Brady et al (2004) ^[S1]^ | Retrospective Cohort | USA | 131 | BSE, VFSS and FEES:  ASHA NOMS  And non-validated: Aspiration  Penetration | Not recorded | No |
| Chang et al (2023) ^[S2]^ | Retrospective  Cohort | Korea | 56 | VFSS: PAS  VDS | Not recorded | No |
| **Study ID** | **Study Design** | **Location** | **No of Participants** | **Outcome measures with instrumentation used to define dysphagia** | **Time post injury of when dysphagia assessment completed** | **Follow up** |
| Hamilton et al (2022) ^[S4]^ | Prospective Cohort | USA | 20 | VFSS | Not recorded | No |
| Hayashi et al (2017) ^[S5]^ | Retrospective Cohort | Japan | 298 | FOIS (instrumentation not documented) | 3 days post injury | No |
| Ihalainen et al (2018) ^[S6]^ | Prospective Cohort | Finland | 37 | VFSS :PAS | 2 weeks of injury  Mean 12. 4 days to VFSS | No |
| Kirshblum et al (1999) ^[S7]^ | Retrospective Cohort | USA | 187 | VFSS: Non-validated: Abnormal result | Not Recorded | No |
| Lee and Shin (2023) ^[S8]^ | Retrospective Cohort | Korea | 149 | VFSS: Non validated Penetration or Aspiration | Not recorded | Yes up to 90 days |
| Meißner et al (2025) ^[S9]^ | Retrospective  Cohort | Germany | 403 | FEES: PAS | Not recorded | No |
| **Study ID** | **Study Design** | **Location** | **No of Participants** | **Outcome measures with instrumentation used to define dysphagia** | **Time post injury of when dysphagia assessment completed** | **Follow up** |
| Miles et al (2021) ^[S10]^ | Prospective  Cohort | New Zealand | 75  (64 with cSCI gained from raw data) | FEES and VFSS: PAS  FOIS  YALE  NZSS | Not recorded | Yes up to 180 days |
| Mitsuishi and Ishihara (2019) ^[S11]^ | Retrospective  Cohort | Japan | 80 | Not reported | Not recorded | Yes 30 and 60 days |
| Seidl et al (2010) ^[S12]^ | Retrospective cohort | Germany | 175 | FEES: Non-validated  No abnormalities  Penetration with cough  Aspiration with Cough  Aspiration without cough | Not recorded | No (but did follow up regarding dysphagia treatment) |

| **Study ID** | **Study Design** | **Location** | **No of Participants** | **Outcome measure with instrumentation used to define dysphagia** | **Time post injury of when dysphagia assessment completed** | **Follow up** |
| --- | --- | --- | --- | --- | --- | --- |
| Shem et al (2005) ^[S13]^ | Retrospective  Cohort | USA | 68 | VFSS and BSE: Non-validated Dysphagia yes or no | Not recorded | Yes not as recorded in days to regular diet with maximum being 88 days |
| Shem et al (2012) ^[S14]^ | Diagnostic accuracy study | USA | 29 | “positive aspiration” on VFSS and BSE. Not validated | Within 72 hours | No |
| Shem et al (2019) ^[S15]^ | Prospective Cohort | USA | 76 | VFSS and Bedside:  Non-validated oral intake scale | 24 hours BSE  72 hours VFSS  (after admission) | No |
| Shin et al (2011) ^[S16]^ | Retrospective cohort | Korea | 121 | VFSS and BSE: non validated | 178 Days (range 12-1062) | No |
| Taguchi et al (2024) ^[S17]^ | Retrospective cohort | Japan | 100 | FEES: Non-validated  Main outcome for dysphagia severity – Full oral nutrition on discharge vs parental nutrition on discharge | Not recorded | No |
| Yoon et al (2019) ^[S18]^ | Cross-sectional | Korea | 17 (raw data) | FEES and VFSS | Not recorded | No |

**Appendix 3 References of Studies included in the Systematic review**

[S1] Brady, S, Miserendino, R, Statkus, D, Springer, T, Hakel, M, Stambolis,

V. Predictors to Dysphagia and Recovery After Cervical Spinal Cord

Injury During Acute Rehabilitation. The Journal of Applied Research. 2004;

4(1): 1-11

[S2] Chang, M, Kim, D, Choi, J, Choi, H, Park, J, Park, D. Association between

anterior surgical approach and dysphagia severity in patients with cervical

spinal cord injury. Journal of Clinical Medicine. 2023; 12(9): 3227

[S3] Choi, J-W, Kim, D.Y, Joo, S. Y, Park, D, Chang, M. Evaluation of

clinical factors predicting dysphagia in patients with traumatic and non-

traumatic cervical spinal cord injury: a retrospective study. Frontiers in

Neurology, 15: 1-6.

[S4] Hamilton, V, Pitts, L, Walaszek, E, Cherney, L. Videofluoroscopic profiles of

swallowing and airway protection post-traumatic cervical spinal cord injury.

Dysphagia. 2022; 37(6): 1599–1611.

[S5] Hayashi, T, Fujiwara, Y, Sakai, H, Maeda, T, Ueta, T and Shiba, K. Risk

factors for severe dysphagia in acute cervical spinal cord injury. Spinal Cord.

2017; 55(10):940-943.

[S6] Ihalainen, T, Rinta-Kiikka, I, Luoto, T.M, Thesleff, T, Helminen, M, Korpijaakko-

Huuhka, A, Ronkainen, A. Risk factors for laryngeal penetration-

aspiration in patients with acute traumatic cervical spinal cord injury. The

Spine Journal. 2018; 18(1): 81–87.

[S7] Kirshblum, S, Johnston, M, Brown, J, O’Connor, K, Jarosz, P. Predictors of

dysphagia after spinal cord injury. Archives of Physical Medicine

and Rehabilitation. 1999; 80(9): 1101–1105.

[S8] Lee, S, Shin, J. Pulmonary function and sarcopenia as

predictors of dysphagia in cervical spinal cord injury. Spinal Cord. 2024; 62(1):

42–49.

[S9] Meißner, I, Dietmann, S, Hüller, G, Mach, O, Vogel, M, Ehret. Risk Factors for

Dysphagia After Traumatic Cervical Spinal Cord Injury: A Retrospective Study.

Archives of Physical Medicine and Rehabilitation. 2025; 106 (8): 1189-1197.

[S10] Miles, A, Jamieson, G, Shasha, L, Davis, K. Characterizing Dysphagia after

spinal surgery. Journal of Spinal Cord Medicine. 2021; 44(5): 733–741

[S11] Mitsuishi, T, Ishihara, Y. Association between Prevertebral Soft

Tissue Thickening and Dysphagia in Cases of Traumatic Cervical Spinal Cord

Injury. Prog Rehabil Med*.* 2019; 1 (4:20190019): 9

[S12] Seidl, R, Nusser-Müller-Busch, R, Kurzweil, M, Niedeggen, A. Dysphagia in

acute tetraplegics: a retrospective study. Spinal Cord. 2010; 48(3): 197-201

[S13] Shem, K, Castillo, K, Naran, B. Factors associated with dysphagia

in individuals with high tetraplegia. Topics in Spinal Cord Injury Rehabilitation.

2005; 10(3): 8–18

[S14] Shem, K, Castillo, K, Wong, S, Chang, J, Kolakowsky-Hayner, S.

Dysphagia and respiratory care in individuals with tetraplegia: incidence,

associated factors, and preventable complications. Topics in Spinal Cord

Injury Rehabilitation. 2012: 18(1): 15–22.

[S15] Shem, K, Wong, J, Dirlikov, B, Castillo, K. Pharyngeal dysphagia

in individuals with cervical spinal cord injury: a prospective observational

cohort study. Topics in Spinal Cord Injury Rehabilitation. 2019; 25(4): 322–

330.

[S16] Shin, J, Yoo, J, Lee, Y, Goo, H, Kim, H. Dysphagia in

cervical spinal cord injury. Spinal Cord*.* 2011; 49(9): 1008–1013.

[S17] Taguchi, K, Obayashi, S, Nishimoto, J, Fujimoto, M. Fiberoptic endoscopic

evaluation of swallowing in patients with cervical spinal cord injury: a

retrospective study focused on oral intake at discharge. Research

Square (Pre Print). 2024. <https://doi.org/10.21203/rs.3.rs-3904457/v1>.

Accessed 3 August 2025.

[S18] Yoon, J, Kim, S, Jang, M, Kim, S, Shin, Y. Correlations

between aspiration and pharyngeal residue scale scores for fiberoptic

endoscopic evaluation and videofluoroscopy. Yonsei Medical Journal. 2019;

60(12): 1181–1186.

**Appendix 4 Summary of descriptive statistics for the risk factors**

| **Risk Factor** | **Number of Studies** | **Those with Dysphagia** | **Those without dysphagia** |
| --- | --- | --- | --- |
| **Age**    **Mean (SD)** | 11  [S2, S3, S5, S6, S7, S8, S9, S10, S11, S15, S18] | 57.74 (28.32) | 50.61 (22.02) |
| **Gender**    **Male n(%)** | 8  [S3, S5, S6, S8, S9, S11, S15,S17] | 323 (29.9%) | 42 (19.4%) |
| **Neurological Level**    **High (C1-C4) n(%)** | 7  [S5, S6, S8, S9, S15, S17, S18] | 207 (33.2%) | 85 (17.6%) |
| **Level of Completeness**  **Complete (ASI A) n(%)** | 8  [S3, S5, S6, S8, S9, S13, S16, S17] | 152 (34.1%) | 203 (30.3%) |
| **Tracheostomy**  **Yes (n%)** | 12  [S1, S3, S5, S6, S7, S9, S10, S11, S15, S16, S17] | 241 (47.9%) | 229 (18.7%) |
| **Surgery**  **Yes n(%)** | 8  [S1, S5, S6, S7, S13, S15, S16, S17] | 169 (30.0%) | 36 (11.9%) |
| **Surgical Approach**  **Anterior n(%)** | 11  [S1, S3, S6, S7, S8, S9, S10, S12, S16, S17] | 268 (33.6%) | 157 (28.4%) |
| **Surgical Approach**  **Posterior (n%)** | 8  [S1, S3, S7, S9, S12, S15, S16, S17] | 121 (34.4%) | 213 (25.4%) |
| **Surgical Approach**  **Combined (n%)** | 4  [S1, S9, 12, S16] | 45 (32.1%) | 157 (22.7%) |
| **FVC (% predicted)**  **Mean (SD**) | 2  [S3, S8] | 44.29 (19.91) | 58.98 (16.83) |
| **Mechanical Ventilation**  **Yes n (%)** | 2  [S15, S17] | 58 (53.7%) | 30 (44.1%) |
| **Aetiology**  **Traumatic n (%)** | 3  [S1, S3, S7] | 80 (31.0%) | 36 (39.1%) |
| **NGT**    **Yes (n%)** | 2  [S15, S17] | 67 (67%) | 21 (27.6%) |
| **Concurrent Brain Injury**  **Yes (n%)** | 2  [S1, S15] | 37 (61.7%) | 58 (39.5%) |
| **Presence of Collar**  **Yes (n%)** | 3  [S1, S12, S15] | 75 (66.7%) | 46 (29.5%) |
| **Presence of Halo**  **Yes(n%)** | 3  [S1, S12, S15] | 40 (32.5%) | 27 (7.7%) |
| **History of Bronchoscopy**  **Yes (n%)** | 2  [S6, S15] | 11 (68.5%) | 29 (33.0%) |
| **History of Pneumonia**  **Yes (n%)** | 2  [S15, S16] | 15 (48.4%) | 16 (10.2%) |

**Appendix 5 Results of Newcastle Ottawa Scale completed for cohort Studies**

| **Author (date)** | **Selection (Max 4)** | | | | **Comparability (Max 2)** | **Outcome (Max 3)** | | | **Total Score** |
| --- | --- | --- | --- | --- | --- | --- | --- | --- | --- |
|  | **1** | **2** | **3** | **4** | **5** | **6** | **7** | **8** |  |
|  | Representativeness of the exposed cohort | Selection of the non-exposed cohort  Selection of the non-exposed cohort | Ascertainment of exposure | Outcome not present at start of study | Comparability of cohorts on confounders (e.g. age, sex) | Assessment of outcome | Was follow-up long enough for outcomes to occur | Adequacy of follow-up of cohorts |  |
| **Brady et al (2004)** | ✓ | 🗶 | ✓ | ✓ | ✓🗶 | ✓ | ✓ | 🗶 | 6/9 Moderate |
| **Chang et al (2023)** | ✓ | ✓ | ✓ | ✓ | ✓✓ | ✓ | ✓ | 🗶 | 8/9 High |
| **Choi et al (2024)** | ✓ | ✓ | ✓ | ✓ | ✓✓ | 🗶 | 🗶 | 🗶 | 6/9 Moderate |
| **Hamilton et al (2022)** | ✓ | 🗶 | ✓ | ✓ | ✓🗶 | ✓ | ✓ | 🗶 | 6/9 Moderate |
| **Hayashi et al (2017)** | ✓ | 🗶 | 🗶 | ✓ | ✓✓ | ✓ | ✓ | 🗶 | 6/9 Moderate |
| **Hayashi et al (2020)** | ✓ | 🗶 | ✓ | **🗶** | ✓✓ | ✓ | ✓ | 🗶 | 6/9 Moderate |
| **Ihalainen et al (2018)** | ✓ | ✓ | ✓ | ✓ | ✓✓ | ✓ | ✓ | 🗶 | 8/9 High (Downgraded to 7/9 due to missing data) |
| **Kirshblum et al (1999)** | ✓ | 🗶 | ✓ | ✓ | ✓✓ | 🗶 | 🗶 | 🗶 | 5/9 Moderate  (Downgraded to 4/9 due to missing data) |
| **Lee and Shin (2024)** | ✓ | ✓ | ✓ | ✓ | ✓✓ | 🗶 | ✓ | ✓ | 8/9 High |
| **Meißner et al (2024)** | ✓ | ✓ | ✓ | ✓ | ✓✓ | ✓ | 🗶 | 🗶 | 7/9 High |
| **Miles et al (2021)** | ✓ | 🗶 | ✓ | 🗶 | ✓🗶 | ✓ | 🗶 | ✓ | 5/9 Moderate |
| **Mitsuishi and Ishihara (2019)** | ✓ | ✓ | 🗶 | 🗶 | ✓✓ | ✓ | ✓ | ✓ | 7/9 High |
| **Seidl et al (2010)** | ✓ | ✓ | ✓ | 🗶 | ✓🗶 | 🗶 | 🗶 | 🗶 | 4/9 Moderate |
| **Shem et al (2005)** | ✓ | ✓ | ✓ | 🗶 | ✓🗶 | 🗶 | 🗶 | ✓ | 5/9 Moderate |
| **Shem et al (2019)** | ✓ | ✓ | ✓ | ✓ | ✓🗶 | 🗶 | ✓ | 🗶 | 6/9 Moderate |
| **Shin et al (2011)** | ✓ | ✓ | ✓ | ✓ | ✓🗶 | 🗶 | 🗶 | 🗶 | 5/9 Moderate |
| **Taguchi et al (2024)** | ✓ | ✓ | ✓ | ✓ | ✓✓ | 🗶 | 🗶 | 🗶 | 6/9 Moderate  (Downgraded to 5/9 as preprint) |

**Appendix 6 - Results table for all risk ratios, odds ratios and mean differences calculated in meta-analysis both multivariate and univariate data for comparison with a GRADE rating for the 6 most clinically relevant risk factors**

| **Risk Factor** | **Multivariate**  **Logistic Regression**  **OR (95% CI) p-value** | **Univariate**  **OR (95% CI) p-value** | **Risk Ratio**  **RR (95% CI) p-value** | **Mean Difference**  **MD (95%) p-value** | **GRADE** |
| --- | --- | --- | --- | --- | --- |
| Tracheostomy  (Yes vs No) | N= 5 (1,028 ppt)  4.91 (1.74 to 13.86) p=0.003 | N= 8 (780 ppt) 3.52 (1.57 to 7.85)  p=0.002 | N= 8 (780 ppt)  2.10 (1.29 to 3.44) p=0.003 | - | ⬤◯◯◯ (Very Low)  Interpret with substantial caution |
| Surgery (Yes vs No) | N=3 (414 ppt)  2.11 (0.63 to 7.08) p=0.23 | N= 9 (1,196 ppt)  1.38 (0.69 to 2.79) p=0.36 | N=9 (1,196 ppt)  1.15 (0.74 to 1.80) p=0.53 | - | N/A |
| Anterior surgical approach | N= 5 (724 ppt)  1.03 (0.26 to 4.09) p= 0.96 | N=11 (1,351 ppt)  1.02 (0.69 to 1.49)  p=0.94 | N=11 (1,351 ppt)  1.03 (0.84 to 1.26) p=0.78 | - | N/A |
| Posterior Surgical Approach | - | N=9 (1,292 ppt)  0.80 (0.47 to 1.34) p=0.40 | N=9 (1,292 ppt)  0.91 (0.66 to 1.24) p=0.54 | - | N/A |
| Combined Surgical Approach | - | N=5 (880 ppt)  1.77 (1.17 to 2.70)  P=0.007 | N=5 (880 ppt)  1.38 (1.10 to 1.74)  P=0.006 | - | ⬤◯◯◯ (Very Low)  Interpret with substantial caution |
| Neurological Level  (High vs Low) | N=3 (850 ppt)  1.31 (0.55 to 3.11)  p=0.54 | N= 4 (229 ppt)  1.38 (0.73 to 2.59)    p=0.32 | N=4 (229 ppt)  1.11 (0.88 to 1.41)  p=0.38 | - | ⬤◯◯◯ (Very Low)  Interpret with substantial caution |
| Completeness of cSCI  (Complete vs Incomplete) | N=2 (186 ppt)  1.07 (0.16 to 7.09) p= 0.94 | N=6 (815 ppt)  1.42 (0.79 to 2.55) p=0.24 | N=6 (815 ppt)  1.25 (0.90 to 1.75) p=0.02 | - | ⬤◯◯◯ (Very Low)  Interpret with substantial caution |
| Gender  (Male vs Female) | N=1 (149 ppt)  1.08 (1.04 to 1.12) p<0.0001 | N=9 (1,295 ppt)  1.95 (1.32 to 2.88)  p=0.0009 | N=9 (1,295 ppt)  1.50 (1.15 to 1.96) p=0.003 |  | ⬤⬤◯◯ (Low)  Interpret with limited confidence |
| Forced Vital Capacity (Continuous) | N=2 (247 ppt)  0.95 (0.86 to 1.04) p=0.28 | - | - | N=2 (247 ppt)  -13.16 (-17.26 to -9.07) p<0.000001 | N/A |
| Aetiology  (Trauma vs Non-Trauma) | - | N=3 (326 ppt)  1.91 (0.84 to 4.36)  P=0.12 | N=3 (326 ppt)  1.56 (0.89 to 2.75)  P=0.12 | - | N/A |
| Age (Continuous) | N=3 (589 ppts)  1.04 (1.00 to 1.08) p=0.07 | - | - | - | N/A |
| Age (Binary <72) | N=1 (298 ppts)  3.45 (1.40 to 8.48) p=0.007 | - | - | N=7 (593 ppts)  3.11 (-0.33 to 6.55) p=0.08 | ⬤◯◯◯ (Very Low)  Interpret with substantial caution |
| Concurrent Brain Injury  (Yes vs No) | - | N=2 (207 ppt)  2.96 (0.93 to 9.45) p=0.07 | N=2 (207 ppt)  1.72 (1.34 to 2.21) p<0.0001 | - | N/A |
| Mechanical Ventilation  (Yes vs No) | - | N=2 (176 ppt)  1.07 (0.16 to 7.31) p=0.94 | N=2 (176 ppt)  1.20 (0.40 to 3.62) p=0.75 | - | N/A |
| Presence of NGT  (Yes vs No) | - | N=2 (176 ppt)  4.99 (1.20 to 20.73) p=0.03 | N=2 (176 ppt)  2.51 (1.30 to 4.83) p=0.006 | - | N/A |
| Bronchoscopy  (Yes vs No) | - | N=2 (104 ppt)  3.87 (1.06 to 14.14) p=0.04 | N=2 (104 ppt)  2.00 (1.30 to 3.07) p=0.002 | - | N/A |
| Presence of Collar (Yes vs No) | - | N=3 (379 ppt)  1.01 (0.63 to 1.64) p=0.94 | N=3 (379 ppt)  1.01 (0.77 to 1.33) p=0.93 | - | N/A |
| Presence of Halo Traction Device (Yes vs No) | - | N=3 (382 ppt)  1.60 (0.76 to 3.37)  P=0.21 | N=3 (382 ppt)  1.31 (0.92 to 1.85)  P=0.13 | - | N/A |
| History of Aspiration (Yes vs No) | N=1 (131 ppt)  6.55 (2.66 to 16.14) p<0.0001 | N=2 (188 ppt)  5.21 (1.89 to 14.32) p=0.001 | N=2 (188 ppt)  3.15 (1.56 to 6.39) p=0.001 | - | N/A |

Foot note: N = number of studies included in the meta-analysis

N/A – GRADE not completed
